# Layer-by-Layer Engineered Zinc–Tin Oxide/Single-Walled Carbon Nanotube (ZTO/SWNT) Hybrid Films for Thin-Film Transistor Applications

**DOI:** 10.3390/mi16070825

**Published:** 2025-07-20

**Authors:** Yong-Jae Kim, Young-Jik Lee, Yeon-Hee Kim, Byung Seong Bae, Woon-Seop Choi

**Affiliations:** Department of Semiconductor Engineering, Hoseo University, Asan 31499, Republic of Korea

**Keywords:** oxide TFT, single-walled carbon nanotubes, layer by layer, hybrid films

## Abstract

Indium-based oxide semiconductors have been commercialized because of their excellent electrical properties, but the high cost, limited availability, and environmental toxicity of indium necessitate the development of alternative materials. Among the most promising candidates, zinc–tin oxide (ZTO) is an indium-free oxide semiconductor with considerable potential, but its relatively low carrier mobility and inherent limitations in thin-film quality demand further performance enhancements. This paper proposes a new approach to overcome these challenges by incorporating single-walled carbon nanotubes (SWNTs) as conductive fillers into the ZTO matrix and using a layer-by-layer multiple coating process to construct nanocomposite thin films. As a result, ZTO/SWNTs (0.07 wt.%) thin-film transistors (TFTs) fabricated with three coating cycles exhibited a high saturation mobility of 18.72 cm^2^/V·s, a threshold voltage of 0.84 V, and a subthreshold swing of 0.51 V/dec. These values represent an approximately four-fold improvement in mobility compared to ZTO TFT, showing that the multiple-coating-based nanocomposite strategy can effectively overcome the fundamental limitations. This study confirms the feasibility of achieving high-performance oxide semiconductor transistors without indium, providing a sustainable pathway for next-generation flexible electronics and display technologies.

## 1. Introduction

Solution-processed oxide semiconductors are an attractive alternative to conventional silicon-based thin-film transistor (TFT) fabrication methods, offering low production cost, excellent compatibility with large-area processing, outstanding mechanical flexibility, and high optical transparency. Various oxide TFTs based on indium oxide (In_2_O_3_) and indium–gallium–zinc oxide (IGZO) have been extensively studied due to their simple solution processability, high carrier mobility, and excellent compatibility with flexible substrates. In particular, recent studies have demonstrated that hybridizing these oxides with single-walled carbon nanotubes (SWNTs) can significantly improve the electrical performance of the TFTs by enhancing carrier transport and reducing defect states [1,2].

Among these solution-processable oxides, zinc–tin oxide (ZTO) is a promising channel material for next-generation high-performance electronics. ZTO exhibits a wide bandgap and excellent chemical stability. More importantly, it eliminates the use of indium, a rare, expensive, and environmentally alarming element. ZTO-based TFTs can be fabricated using various techniques, including solution coating, inkjet printing, and electrohydrodynamic jet printing, and they show excellent compatibility under low-temperature processing conditions [3,4]. Despite their simplified processing and scalability advantages, ZTO TFTs fabricated at low temperatures often suffer from poor mobility and instability. These issues have been attributed primarily to structural disorder, compositional non-uniformity, and oxygen-related defects, which remain critical challenges for further performance improvement [5]. Therefore, strategies for enhancing material quality and device architecture are necessary.

The integration of high-mobility, one-dimensional nanomaterials, such as single-walled carbon nanotubes (SWNTs), to address the aforementioned limitations has attracted increasing interest. SWNTs have exceptional electrical conductivity, a high aspect ratio, and remarkable mechanical flexibility. They can serve as auxiliary charge transport pathways within oxide semiconductor channels, contributing to reduced trap densities and enhanced structural integrity of the thin films. The pursuit of indium-free, solution-processed oxide semiconductors capable of delivering high performance and scalability remains an unresolved challenge.

The authors’ research group proposed two hybrid strategies to overcome the inherent limitations of ZTO by incorporating SWNTs into the ZTO matrix. The first approach involved blending SWNTs directly into the ZTO precursor solution to form a single-layer hybrid thin film. The introduction of SWNTs effectively supplemented the charge transport pathways, enhancing carrier mobility [6]. The second approach involved fabricating a layered hybrid structure by sequentially depositing ZTO and SWNT layers. In particular, the top ZTO/bottom SWNT configuration exhibited the most favorable electrical performance, suggesting that positioning SWNTs at the bottom facilitates more efficient electron transport [7].

Both studies showed that incorporating SWNTs improved electrical performance compared to pristine ZTO-based devices. On the other hand, these earlier studies focused primarily on single-layer or simple bilayer configurations, which posed limitations in achieving structural consistency and processing stability. In particular, the issues of precise control over interlayer structures during solution processing remain critical. These factors can affect electrical uniformity and compromise device reliability [8].

In response to the limitations of past approaches, this study proposed a new hybrid thin-film fabrication strategy that integrates layer-by-layer deposition with surface treatment techniques. This approach enables precise control over the formation of each layer, promoting continuous charge transport pathways, improving process reproducibility, and promoting a uniform thin-film morphology. As a result, the ZTO/SWNT composite thin films developed in this study exhibit superior field-effect mobility, reduced off-state current, and enhanced device stability compared to those fabricated using conventional methods. These improvements validate the effectiveness of optimizing the material’s composition and the thin-film architecture. This method establishes a scalable and practical platform for indium-free oxide semiconductors, paving the way for next-generation, high-performance electronics.

## 2. Experimental

The sol–gel precursors for active channel layer formation were prepared by dissolving zinc acetate dihydrate and tin(II) chloride dihydrate in 2-methoxyethanol. The effects of the tin content on thin-film characteristics were examined by fixing the molar ratio of zinc to three while systematically varying the molar ratio of tin from one to five. Each precursor solution was stirred at 50 °C for 24 h to ensure complete dissolution and homogeneous mixing.

SWNTs with an average diameter and length of ~1.1 nm and 5–30 μm (purity > 90 wt.%, US Research Nanomaterials, Inc., Houston, TX, USA), respectively, were dispersed in 2-methoxyethanol and ultrasonicated at 20 °C for one hour to ensure stable dispersion. Based on previous optimization studies [7], the SWNT’s concentration was fixed to 0.07 wt.% relative to the total metal precursor content. The resulting ZTO/SWNT hybrid solution was passed through a 5 μm syringe filter to remove any aggregates prior to coating.

**Figure 1:**
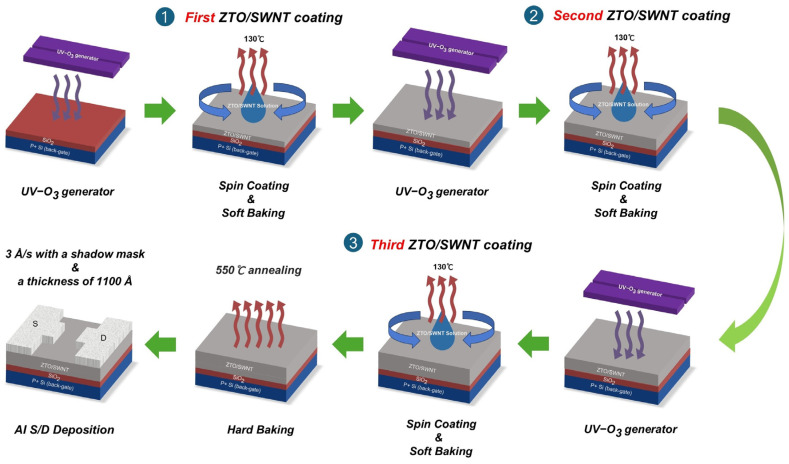
Schematic diagram of the fabrication process for solution-processed ZTO/SWNT hybrid three-layered thin film and its transistor.

Thin-film fabrication was performed using a layer-by-layer deposition strategy, as shown in Figure 1. Heavily doped p-type silicon substrates with a 300 nm thermally grown SiO_2_ layer were pre-cleaned and treated with UV/ozone (254 nm, 20–30 mW cm^−2^) for 30 min to enhance the surface hydrophilicity and promote interfacial adhesion. The ZTO/SWNT precursor solution was spin-coated at 5500 rpm for 60 s, followed by soft-baking at 130 °C for 15 min to remove the residual solvents. Before depositing each subsequent layer, the film surface was reactivated by UV/ozone exposure to restore the surface energy and ensure strong interlayer contact. This layer-by-layer sequence resulted in various film thicknesses depending on the number of coating cycles. The films were thermally annealed at 550 °C for 80 min in ambient air to densify the oxide matrix and reduce defect states.

Bottom-gate, top-contact TFTs were fabricated by thermally evaporating the aluminum (Al) source and drain electrodes (thickness: 1100 Å) through a patterned shadow mask with a channel width and length of 1500 μm and 100 μm, respectively. Electrical measurements were carried out under dark conditions at room temperature using a semiconductor parameter analyzer (Keithley 4200A-SCS, Keithley Instruments, Solon, OH, USA). All electrical characteristics were measured from fifteen independently fabricated devices (*n* = 15) for each coating condition, and the data represent average values along with their variations.

Subsequently, a comprehensive set of structural and compositional analyses was conducted to elucidate the microstructure and interfacial characteristics of the hybrid films. Scanning electron microscopy (SEM; MAIA3, TESCAN, Brno, Czech Republic) was used to investigate the surface morphology, while energy-dispersive X-ray spectroscopy (EDS; X-MaxN, Oxford Instruments, High Wycombe, UK) was performed in plan-view and cross-sectional geometries to quantify the spatial distribution of the key elements. X-ray photoelectron spectroscopy (XPS; K-Alpha, Thermo Fisher Scientific, East Grinstead, UK) utilizing an Al Kα source (1486.6 eV) revealed the chemical states of Zn, Sn, O, and C species, with particular emphasis on the interfacial interactions between the ZTO matrix and embedded SWNTs. Atomic force microscopy (AFM; NX10, Park Systems, Suwon, Republic of Korea) in tapping mode was used to examine the surface topography and roughness of the films.

## 3. Results and Discussion

Previous studies have reported that hybridization of ZTO with SWNTs enables the nanotubes to act as effective charge transport pathways, enhancing the carrier mobility and reducing the off-state current [6,7]. Precise control of the SWNT’s concentration showed that optimal electrical performance was achieved at 0.07 wt.%. At higher concentrations, however, the device’s performance deteriorated because of aggregation and interfacial inhomogeneity. These findings suggest that single-layer or conventionally stacked configurations impose inherent structural and process-related limitations in finely controlling the interactions between the composite components. The dispersion state of SWNTs, their interfacial chemistry, and the thermal and chemical interactions that occur during layer-by-layer fabrication have nonlinear and interdependent effects on the device’s performance. Such complexity underscores the need for advanced structural control strategies to manage these intertwined variables precisely.

This study introduced a newly designed layer-by-layer multiple coating process for fabricating ZTO composite thin films incorporating SWNTs to overcome the aforementioned limitations. Integrating interfacial control and thermal treatment at each deposition step enhanced structural densification and surface reactivity while improving reproducibility and ensuring film uniformity. In particular, the precise stacking control enabled by the multilayer architecture is expected to promote the continuity of the charge transport pathways. This structural refinement provides a favorable foundation for enhancing the electrical performance of the resulting devices.

A previous study optimized the Zn:Sn molar ratio in solution-processed pure ZTO thin-film transistors and found that a 1:1 composition yielded the highest field-effect mobility along with minimized leakage current [9]. Nevertheless, these results were derived from a single-phase system without SWNTs, which may not directly translate to the behavior of the ZTO/SWNT hybrid system. In composite oxide channels, inter-material interactions can significantly influence properties like grain growth orientation, interfacial trap density, and the continuity of charge transport pathways. As a result, the impact on device performance may differ considerably, even with identical chemical compositions [10]. The first study aims to address this by elucidating the relationship between composition, structure, and electrical properties within the ZTO/SWNT hybrid system by varying the Zn:Sn molar ratio while fixing the SWNT concentration at 0.07 wt.%.

**Figure 2:**
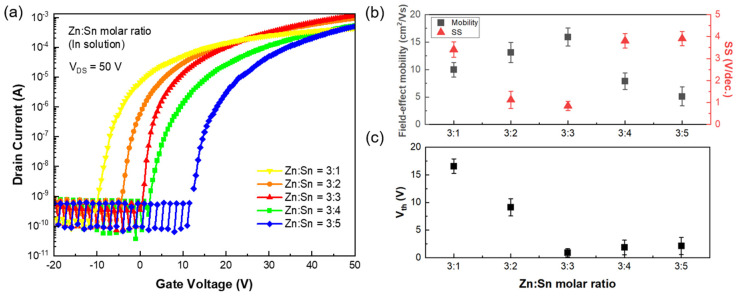
Electrical characteristics of ZTO/SWNT hybrid thin films with various Zn:Sn molar ratios. (**a**) Transfer curves (at V_ds_ = 50 V), (**b**) field-effect mobility and subthreshold slope, and (**c**) threshold voltage (Vth) variation as a function of the Zn:Sn molar ratio.

The electrical characteristics of the corresponding TFTs (Figure 2a–c) quantitatively reflect the influence of the Sn content on charge carrier density and interfacial energetics within the channel layer. The threshold voltage (Vth) shifted progressively from positive to negative as the Sn concentration increased, indicating that an enhanced free carrier population in the oxide matrix modulates the electrostatic potential landscape of the channel [11]. The field-effect mobility (*μ*_FE_) and subthreshold swing (*SS*) metrics in Figure 2b were maximized at a Zn:Sn ratio of 3:3, where a peak mobility of 14.99 ± 0.56 cm^2^/V·s and a minimized *SS* were observed. These characteristics were attributed to the formation of continuous charge transport pathways and electronically uniform interfaces.

The device’s performance deteriorated sharply when the Sn content exceeded a Zn:Sn ratio of 3:4. This degradation was attributed to the high hydrolytic reactivity of the Sn precursor, which induced localized phase separation and disrupted crystallinity during the sol–gel transition [12]. Such structural inhomogeneities likely led to carrier trapping within the channel and an increased density of interface states at the SiO_2_ gate dielectric, resulting in enhanced leakage currents and degraded switching behavior [13]. The negative shift in Vth and increased channel conductivity observed at high Sn concentrations (Figure 2c) were attributed to the metallic-like electronic states and excess free carriers introduced by excessive Sn incorporation [14].

After preliminary identification of Zn:Sn = 3:3, this study examined how systematic control over film thickness and microstructure influences device characteristics under this fixed ratio. Accordingly, a layer-by-layer deposition strategy was used, enabling precise modulation of the film’s architecture through incremental stacking. The thickness and the density of the embedded SWNT network increased as the number of layers increased, leading to extended and more continuous charge transport pathways. These structural evolutions translated into significant improvements in charge carrier mobility and switching behavior.

**Figure 3:**
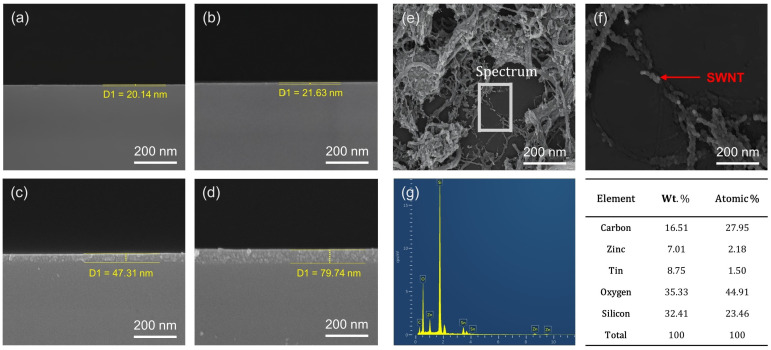
SEM images and EDS analysis of ZTO/SWNT thin films. (**a**–**d**) Cross-sectional SEM images showing a gradual increase in film thickness as the number of layer-by-layer coatings increased: (**a**) pristine ZTO, (**b**) one layer, (**c**) three layers, (**d**) five layers. (**e**) Plan-view SEM image highlighting well-developed rod-like SWNT networks embedded in the ZTO matrix. (**f**) Corresponding wide-area SEM image indicating the EDS analysis region and the uniform dispersion of the nanotube network. (**g**) EDS point spectrum and quantitative elemental composition measured at the SWNT-rich region shown in (**f**).

Figure 3 provides a comprehensive overview of the changes in film thickness and microstructural evolution in ZTO/SWNT thin films fabricated using a layer-by-layer deposition strategy, as shown with SEM, with EDS analysis. The cross-sectional SEM images (Figure 3a–d) revealed a progressive increase in film thickness with the number of coating cycles, showing the conformal and uniform stacking behavior characteristic of the layer-by-layer strategy. Figure 3e,f present top-view SEM images of the film produced with three coating cycles, confirming the presence and distribution of the SWNT network within the hybrid structure. Figure 3e presents the overall morphology, showing the homogeneous dispersion of nanotubes across the surface and indicating the region where EDS analysis was performed. Figure 3f provides a magnified view of this region, where rod-like conductive features corresponding to well-organized SWNT networks are visible. These interconnected nanotubes form a robust percolation structure that enables long-range charge conduction throughout the film. According to percolation theory, a continuous pathway for charge transport spontaneously forms when the density of conductive elements surpasses a critical threshold. The films fabricated in this study appear to exceed this threshold effectively through layer-by-layer deposition [15].

Figure 3g presents the EDS point spectrum acquired from the SWNT-rich region, with the corresponding elemental compositions provided in weight and atomic percentages. In particular, the carbon content reached 16.51 wt.%, approximately 2.4 times higher than the 6.86 wt.% reported for previously stacked ZTO/SWNT films [7]. This increase highlights the enhanced incorporation efficiency and network density of SWNTs achieved using the layer-by-layer strategy, which is believed to play a critical role in improving the charge transport characteristics of the resulting devices.

**Figure 4:**
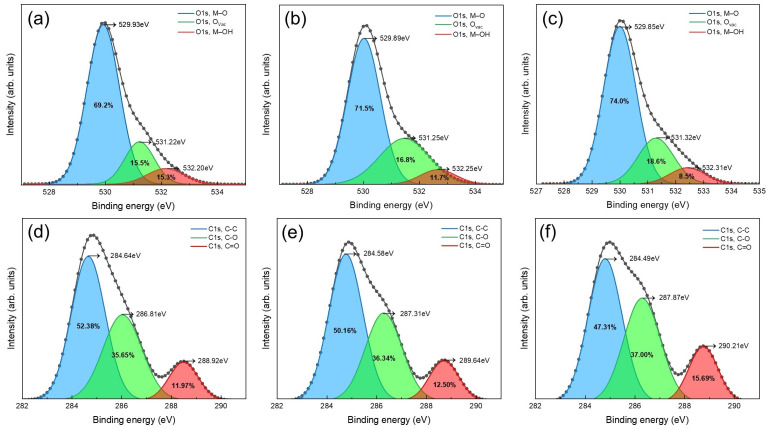
High-resolution XPS spectra of O 1s and C 1s for ZTO/SWNT thin films. (**a**–**c**) O 1s and (**d**–**f**) C 1s spectra of pristine ZTO (**a**,**d**), ZTO/SWNT (one layer) (**b**,**e**), and ZTO/SWNT (three layers) (**c**,**f**) films.

Figure 4 shows the high-resolution XPS spectra of the O 1s (a–c) and C 1s (d–f) regions for a single-layer ZTO film (a,d), a single-coated ZTO/SWNT composite film (b,e), and a triple-layer ZTO/SWNT film fabricated using a layer-by-layer strategy (c,f). The O 1s spectra (a–c) revealed an increase in the peak corresponding to lattice oxygen (M–O) from 69.2% to 74.0% as the number of deposited layers increased, indicating the formation of a denser and more crystalline oxide framework. This densification contributes to reduced interfacial trap density and improved stability of charge transport pathways.

The oxygen vacancy (O_vac_) component increased gradually from 15.5% to 16.8% and 18.6%, suggesting that incorporating SWNTs promotes oxygen release from the ZnO/SnO lattice. The generated vacancies synergize with carbonaceous species to enhance the conduction pathways [16]. Oxygen vacancies can act as shallow donors that provide free electrons to the conduction band, improving n-type conductivity, enhancing the carrier’s mobility, and modulating the electronic structure at the channel interface [17]. Consequently, they are crucial in boosting conductivity and tuning the threshold voltage. The proportion of hydroxyl or organic oxygen species (M–OH) decreased significantly from 15.3% to 8.5%, reflecting the effective suppression of surface hydroxyl groups through the layer-by-layer processing steps.

The C 1s spectra (d–f) showed that C–C bonding was the dominant component in the pristine ZTO film (52.38%), associated primarily with non-conductive sp^2^ carbon. The C–C fraction decreased to 47.31% with layer-by-layer fabrication. In contrast, oxidized carbon species, such as C–O and C=O, increased from 35.65% to 37.00% and from 11.97% to 15.69%, respectively. These variations indicate interfacial reactions and surface oxidation involving oxygen-containing functional groups (–COOH and –OH) on the SWNT surfaces, which may enhance interfacial adhesion and charge injection stability [18].

Table 1 lists the compositional differences among the single-layer ZTO, SWNT-mixed, and layer-by-layer composite films. SWNT incorporation contributes to interfacial oxide purification and facilitates intentional oxygen vacancy generation. Such defect-engineering effects are well-documented as beneficial in transparent and flexible electronic devices [19], especially for achieving stable electrical performance under low- or room-temperature processing conditions. Therefore, the ZTO/SWNT layer-by-layer coating strategy presents a promising approach for the scalable fabrication of next-generation oxide electronics.

**Figure 5:**
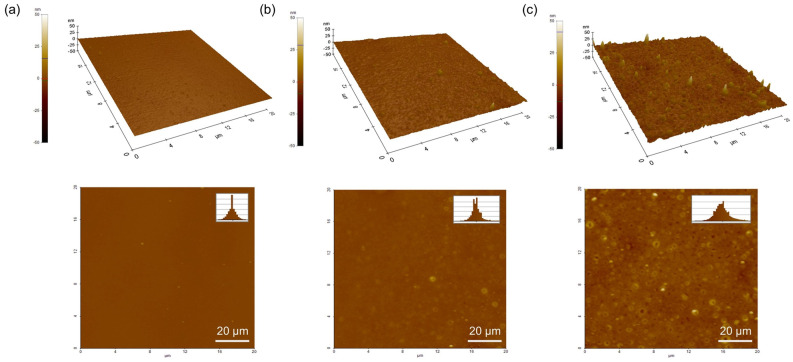
Surface morphologies of ZTO/SWNT thin films with increasing coating cycles. 3D and 2D AFM images of (**a**) pristine ZTO, (**b**) one-time coated ZTO/SWNT, and (**c**) three-time coated ZTO/SWNT films with a 20 × 20 μm^2^ scan area.

Figure 5 presents the three-dimensional and two-dimensional surface topographies of (a) the pristine ZTO single layer, (b) the single-layer ZTO/SWNT composite, and (c) the triple-layer composite film, characterized by atomic force microscopy (AFM). The pristine ZTO film exhibited a relatively smooth and uniform surface, whereas the surface roughness (Ra) of the ZTO/SWNT composite films increased with the number of deposited layers measured at 0.241 nm, 0.742 nm, and 2.039 nm for one, three, and five layers, respectively. This progressive roughening was attributed to the accumulation of nanoscale structural features associated with the incorporation of SWNTs. Previous AFM-based investigations have shown that introducing carbon nanotubes leads to significant surface nano-structuring, typically resulting in a 70–200% increase in roughness [20]. These findings are consistent with the current observations, suggesting that the forming interconnected SWNT networks significantly modulate the surface morphology.

The carrier mobility and electrical stability improved significantly, even with increased roughness resulting from the layer-by-layer construction. Hence, surface roughness alone is not the dominant factor influencing the device’s performance. Instead, the enhanced film densification and the development of continuous charge transport pathways of SWNTs are likely the primary contributors to the observed improvements in the electrical characteristics. In particular, the triple-layer composite film showed a densely packed surface morphology with minimal interfacial voids, indicative of a highly interconnected SWNT network with a local packing density exceeding 90%. This dense architecture supports the formation of continuous carrier transfer rod pathways, minimizing charge scattering and ensuring the integrity of the conductive network. Such structural refinement is pivotal in enhancing carrier transport and device reliability [21].

**Figure 6:**
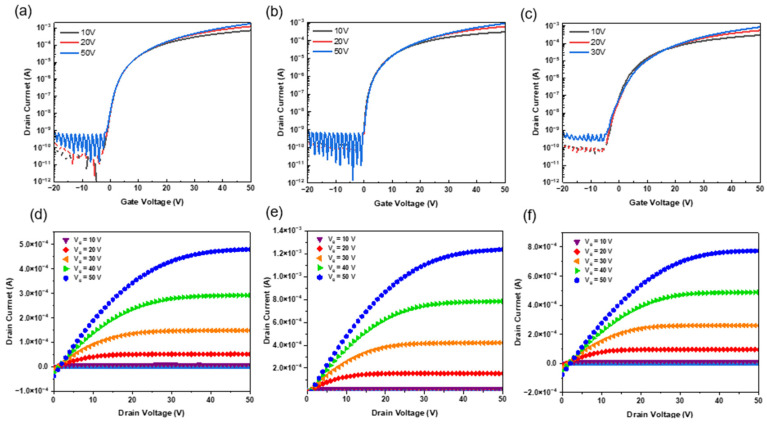
Transfer characteristics and output characteristics of ZTO-only and ZTO/SWNT (0.07 wt.%) TFTs with various coating cycles. (**a**,**d**) ZTO only, (**b**,**e**) three-time-coated ZTO/SWNT, (**c**,**f**) five-time-coated ZTO/SWNTs.

Figure 6d–f present the output characteristics of ZTO/SWNT hybrid TFTs measured under various gate voltages. The device fabricated with three coating cycles exhibited superior linear and saturation regimes, indicating minimized interfacial contact resistance between the ZTO matrix and the SWNT network, and enhanced structural coherence within the channel. The drain current increased progressively as the number of coating cycles increased, reflecting the gradual formation and expansion of charge transport pathways throughout the hybrid channel. On the other hand, when the number of layers exceeded three, the excessive film thickness led to increased off-state leakage current and a decline in carrier mobility, ultimately degrading the device’s performance. These findings underscore a critical trade-off between channel thickness and charge transport efficiency in multilayered systems, highlighting the importance of structural optimization. This trend can be consistently observed in the transfer and output curves in Figure 6, which together offer a comprehensive depiction of how the hybrid channel architecture governs the electrical behavior of the devices. Table 2 lists the quantitative electrical parameters for each device configuration.

**Figure 7:**
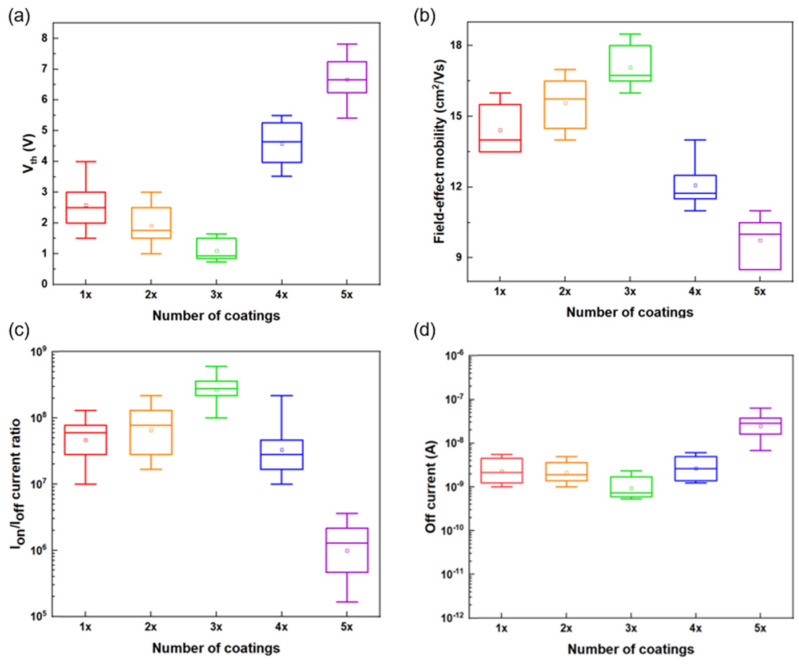
(**a**) Vth vs. SWNT concentrations. (**b**) Field-effect mobility vs. SWNT concentrations. (**c**) I_on_/I_off_ current ratio vs. SWNT concentrations. (**d**) Off current vs. SWNT concentrations of the ZTO/SWNTs nanocomposited TFTs. (n = 15 at each SWNT concentration).

Figure 7a shows the variation in Vth with respect to the number of coating cycles. From one to three coatings, Vth decreased gradually from 1.12 ± 0.08 V to 0.84 ± 0.05 V, which was attributed to the increased carrier concentration in the channel and effective trap passivation induced by the incorporation of metallic SWNTs [22]. Nevertheless, Vth began to rise again beyond three coating cycles, reaching 2.21 ± 0.13 V at five coatings. This reversal was attributed to excessive film thickness and inhomogeneity in the SWNT network, which weaken the effectiveness of the gate electric field [23].

As shown in Figure 7b, *μ* reached a maximum of 18.72 ± 0.56 cm^2^/V·s at three coating cycles. This represents an approximately four-fold improvement compared to the SWNT-free ZTO device (μ = 4.61 ± 0.21 cm^2^/V·s), indicating that incorporating the SWNT network effectively enhanced the formation and continuity of charge transport pathways. The interconnected nanotube framework mitigates structural inhomogeneity and suppresses charge scattering, facilitating continuous conduction channels that improve carrier transport and device reliability [24]. Beyond three coatings, however, the mobility declined because of increased carrier scattering caused by stacking irregularities and the formation of porous aggregates, which also weakened gate field control.

The I_on_/I_off_ current ratio (Figure 7c) exhibited ideal charge-blocking behavior (>10^8^) at three coating cycles. Concurrently, the *SS* reached its lowest value of 0.51 ± 0.03 V/dec, suggesting high charge responsiveness to the gate field and minimal trap-state density. Beyond three coatings, however, the *SS* increased significantly, leading to degraded switching characteristics. This degradation was attributed to SWNT clustering and a rise in trap states, which hindered gate modulation efficiency and degraded the subthreshold behavior [25].

The off-state current decreased gradually up to three coating cycles but increased sharply thereafter, as shown in Figure 7d. This rise was attributed to vertical leakage and trap-assisted tunneling phenomena, which become more pronounced in thicker channels. The underlying causes include increased trap density and non-uniform vertical electric field distribution, which contribute to the degradation of subthreshold leakage characteristics in the device [26].

**Figure 8:**
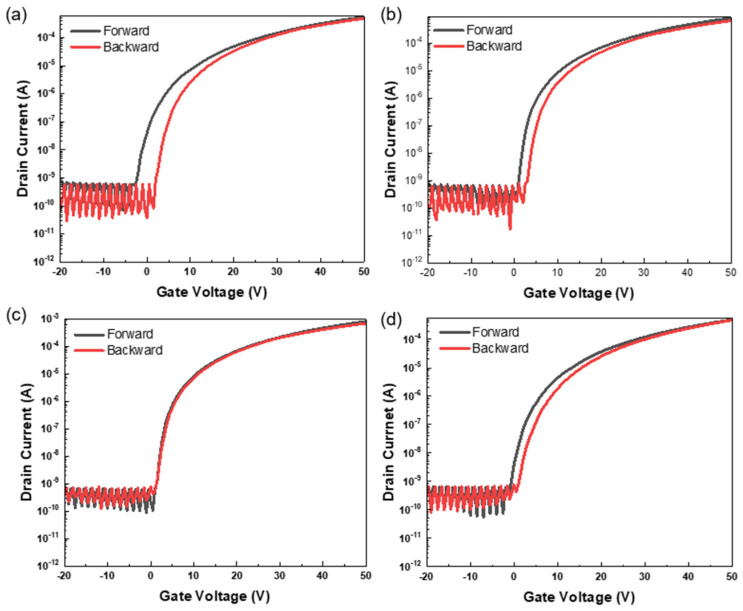
Hysteresis characteristics of ZTO-only and ZTO/SWNT (0.07 wt.%) TFTs with varying spin-coating cycles. (**a**) ZTO only, (**b**) one-time-coated ZTO/SWNTs, (**c**) three-time-coated ZTO/SWNTs, (**d**) five-time-coated ZTO/SWNTs.

The hysteresis behavior of the ZTO/SWNT hybrid TFTs was evaluated by sweeping the gate voltage forward and backward within the range of ±50 V, as shown in Figure 8a–d. All devices showed clockwise hysteresis loops, which were attributed primarily to interface traps located between the SWNT network and the gate dielectric, as well as charge trapping induced by adsorbed water and oxygen molecules on the surface of the ZTO/SWNT channel. This type of clockwise hysteresis is closely associated with electron trapping either within the oxide semiconductor channel or at the oxide–dielectric interface [27].

Among all of the configurations, the device with three coating cycles (Figure 8c) showed the smallest hysteresis voltage shift of approximately 0.07 ± 0.02 V, which was substantially lower than that of the pristine ZTO device (0.72 ± 0.26 V) as well as those with one (0.47 ± 0.16 V) and five (0.52 ± 0.14 V) coatings and even lower than the previously reported stacked structure (0.15 V). These results suggest that the interfacial trap density and internal defect states are effectively suppressed at the optimal composite thickness, minimizing charge trapping phenomena. Furthermore, the repeated thermal annealing process is believed to reduce moisture and oxygen adsorption on the SWNT network’s surface, limiting charge injection and suppressing hysteresis. Simultaneously, the annealing process helps compensate for oxygen vacancies (O_vac_) within the ZTO/SWNT layers, playing a key role in improving the electrical stability of the device.

The layer-by-layer ZTO/SWNT composite structure developed in this study showed enhanced electrical stability and effective hysteresis suppression. This outcome represents a significant technological advance over previously reported stacked configurations [8], offering superior structural uniformity and device reliability. These improvements were attributed to the progressive interconnection and spatial continuity of the SWNT network enabled by the layer-by-layer deposition strategy. A sufficient amount of SWNTs was gradually introduced into the channel by increasing the number of coating cycles, establishing a dense and effective percolation network beyond the critical threshold. In this configuration, the SWNTs functioned as carrier transfer rods, stabilizing the charge transport pathways and bridging spatially heterogeneous conduction regions. Hence, the electrical performance of the device improved dramatically. Moreover, the triple-coating structure presented herein emerged as the optimal design point, overcoming the limitations of conventional mixed and stacked configurations.

## 4. Conclusions

This study developed a uniform and stable sol–gel-based nanocomposite thin film to achieve indium-free next-generation electronics by directly blending ZTO with SWNTs without additional dispersants. Precise control of the nanoscale architecture and interfacial composition was achieved using a layer-by-layer sequential coating strategy, ensuring high fabrication fidelity and reproducibility. The resulting ZTO/SWNT composite transistor exhibited exceptional electrical performance, with field-effect mobility, threshold voltage, and a subthreshold swing of 18.72 ± 0.56 cm^2^/V·s, 0.84 ± 0.05 V, and 0.51 ± 0.03 V/dec, respectively. These values exceeded those of its single-layer and conventionally stacked counterparts based on the same material system, underscoring the effectiveness of the proposed methodology. The layer-by-layer approach enabled high-quality film formation through nanoscale interlayer control, marking a substantial advance in hybrid materials engineering. This strategy provides a robust platform for modulating the formation and dimensional continuity of SWNT percolation networks, enhancing electrical stability, minimizing device-to-device variability, and making it a promising platform for future flexible and wearable electronics.

## Figures and Tables

**Table 1:** Summary of O1s and C1s chemical composition in ZTO/SWNT thin films from XPS analyses.

Sample	O1s (at %)	M-O (% Area)	Vacancy (% Area)	M-OH (% Area)	C-C (% Area)	C-O (% Area)	C=O (% Area)	Total C1s (at %)
ZTO	55.69	69.2	15.5	15.3	52.38	35.65	13.97	12.30
ZTO + SWNT	57.40	71.5	16.8	11.7	50.16	36.34	14.50	11.42
ZTO + SWNT (x3)	59.22	74.0	18.1	8.5	48.31	37.00	15.59	10.93

**Table 2:** Electrical characteristics of ZTO/SWNT hybrid TFTs with various coating cycles.

Device Configuration	$\mu_{\mathrm{sat}}\;({\mathrm{cm}}^{2}/V\cdot s)$	$I_{\mathrm{on}/\mathrm{off}}$	$V_{\mathrm{th}}$ (V)	SS (V/decade)
Pristine ZTO	4.61 ± 0.21	$(2.62\pm 0.15)\times 10^{4}$	13.91 ± 0.62	1.91 ± 0.09
1-cycle coating	16.37 ± 0.42	$(5.41\pm 0.31)\times 10^{7}$	1.12 ± 0.08	0.74 ± 0.04
2-cycle coating	17.24 ± 0.44	$(1.12\pm 0.05)\times 10^{8}$	1.07 ± 0.06	0.63 ± 0.04
3-cycle coating	18.72 ± 0.56	$(7.23\pm 0.32)\times 10^{8}$	0.84 ± 0.05	0.51 ± 0.03
4-cycle coating	14.12 ± 0.38	$(8.21\pm 0.37)\times 10^{7}$	1.24 ± 0.07	1.01 ± 0.06
5-cycle coating	12.23 ± 0.49	$(2.83\pm 0.14)\times 10^{7}$	2.21 ± 0.13	1.22 ± 0.08

## Data Availability

The original contributions presented in this study are included in the article. Further inquiries can be directed to the corresponding author.
